# Post‐Copulatory Sexual Selection in an Insect With High Levels of Mating Failure

**DOI:** 10.1002/ece3.70407

**Published:** 2024-10-16

**Authors:** Vicki L. Balfour, Mélissa Armand, David M. Shuker

**Affiliations:** ^1^ School of Biology University of St Andrews St Andrews UK; ^2^ Animal Comparative Economics Laboratory, Department of Zoology and Evolutionary Biology University of Regensburg Regensburg Germany

**Keywords:** cryptic male choice, *Lygaeus simulans*, mate choice, mating failure, post‐copulatory sexual selection, sperm competition

## Abstract

Sexual selection is not a single process. Instead, multiple processes of sexual selection can interact with respect to a given phenotype, in either reinforcing, independent, or conflicting directions. Here we consider how different processes of sexual selection interact in the seed bug *Lygaeus simulans*. This species is characterised by limited pre‐copulatory sexual selection, but the potential for rather strong post‐copulatory sexual selection. In particular, mating failure is common in this species, with around 40%–60% of copulations failing to result in the successful transfer of sperm. Mating failure is negatively correlated with female size, with smaller females being less likely to end up inseminated. We have recently argued that this pattern is best explained by cryptic male mate choice for large, more fecund females. Males therefore preferentially inseminate larger females. Here we explore how this potential cryptic male choice interacts with another component of post‐copulatory sexual selection: sperm competition. We first manipulated male and female size variation, generating large and small, male and female, size classes. Using a visible mutant marker to assign paternity, we then double‐mated females with males, in all combinations of male and female size. Our results showed that sperm competition outcomes were primarily driven by copulation duration, with longer copulations leading to greater paternity share for a male. We also confirmed that larger females are more likely to produce offspring than smaller females, as predicted by cryptic male choice for large females. This effect was again linked to copulation duration, with longer copulations less likely to lead to mating failure. While larger males tended to be more successful in sperm competition, especially if copulating second, female size had little effect on paternity, suggesting that cryptic male choice and sperm competition are acting relatively independently in this species.

## Graphical abstract

Here we consider how different processes of post‐copulatory sexual selection interact in the seed bug *Lygaeus simulans*. We found that sperm competition outcomes were primarily driven by copulation duration, while larger females were more likely to receive sperm than smaller females, as predicted by cryptic male choice for large females. However, female size had little effect on paternity outcome in our double‐mating experiment, suggesting that cryptic male choice and sperm competition are acting relatively independently in this species.

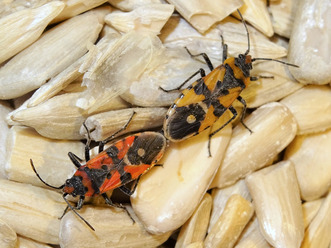

## 1 Introduction

Processes of sexual selection rarely act alone. Scramble competition, contest competition, endurance rivalry and mate choice, can all interact together, sometimes reinforcing one another in terms of the direction of selection, sometimes opposing one another (Andersson 1994; Moore and Moore 1999; Moore et al. 2001; Hunt et al. 2009; Vilella‐Pacheco et al. 2021; Mitchell et al. 2023). Understanding how sexual selection shapes phenotypes therefore requires us to understand how different components of sexual selection act together (Wong and Candolin 2005; Hunt et al. 2009; Shuker 2014). Importantly, both reinforcing and conflicting patterns of sexual selection can occur in terms of both pre‐copulatory and post‐copulatory sexual selection. Indeed, it was the realisation that male–male sperm competition does not act independently of females that has helped galvanise our understanding of the role females play in sexual selection, even if many studies still remain male focused (Thornhill 1983; Eberhard 1996; Cunningham and Birkhead 1998; Simmons 2001; Rosvall 2011; Ah‐King, Barron, and Herberstein 2014; Arnqvist 2014; Ramm and Stockley 2014; Firman et al. 2017; Hare and Simmons 2019, 2020; Sloan and Simmons 2019; Orbach 2022).

Here we consider both sperm competition and cryptic mate choice in the seed bug *Lygaeus simulans*. Males and females of this species mate multiply, and generally pre‐copulatory mate choice is rather limited, with larger females and sometimes larger males being more successful at gaining matings, but not consistently so (Dougherty et al. 2015; Dougherty and Shuker 2016; Balfour, Black, and Shuker 2020; Balfour, Armand, and Shuker 2024). Post‐copulatory sexual selection seems to be more important, however. First, a remarkably high proportion of copulations in which there is sufficient time for genital deployment to occur and ejaculation to commence (30 min or longer) do not involve successful sperm transfer: this so‐called ‘mating failure’ occurs in 40%–60% of copulations (Tadler 1999; Tadler, Nemeschkal, and Pass 1999; Micholitsch, Krügel, and Pass 2000; Greenway and Shuker 2015; Greenway, Balfour, and Shuker 2017; Balfour, Black, and Shuker 2020). Mating failure can arise in a number of ways, from an individual failing to find a mate at all during its life, through to apparently successfully copulations that actually fail to involve sperm transfer (for reviews see Rhainds 2010, 2019; Greenway, Dougherty, and Shuker 2015). Mating failure that occurs despite seemingly successful engagement of genitalia and the attainment of the relevant copulatory position has been called ‘cryptic mating failure’ (Greenway, Dougherty, and Shuker 2015). Crucially, the occurrence of cryptic mating failure in *L*. *simulans* is strongly associated with a lack of sperm transfer, as over 90% of females which failed to produce offspring after a copulation event had no sperm present in their spermatheca (Greenway, Balfour, and Shuker 2017).

In *L. simulans*, such high levels of mating failure do not look to be caused by mechanical failure or incompatibility, nor by genetic incompatibility (Balfour, Armand, and Shuker 2024), but instead by males either choosing not to pass sperm, or females choosing not to receive it (Greenway, Balfour, and Shuker 2017). The most consistent trait associated with mating failure is female size, with larger females more likely to receive sperm, and we have recently argued that this is an example of cryptic male choice for larger and more fecund females (Dougherty and Shuker 2016; Balfour, Black, and Shuker 2020; Balfour, Armand, and Shuker 2024). Mating failure in animals is probably more common than appreciated. For instance, in laboratory studies, pairs that fail to either mate as expected or produce offspring may be discarded, whereas in the field, observations may be biased towards successfully breeding individuals, either at nest sites, breeding colonies, or oviposition sites (Eberhard 1996; Rhainds 2010, 2019; Greenway, Dougherty, and Shuker 2015).

Sperm competition has also been well characterised in this species (Balfour, Black, and Shuker 2020; see also Sillén‐Tullberg 1981 for an earlier paper on the sibling species *Lygaeus equestris*). The data suggest that sperm competition occurs largely via a fair raffle, with copulation duration of both the first and second male largely determining the amount of paternity that each male attains, i.e., the male which copulates for a greater length of time gains a greater share of the paternity (calculated as the proportion sired by the first male [*P*_1_] or second male [*P*_2_] respectively: Balfour, Black, and Shuker (2020). Mating failure of course plays a part here as well though. Balfour, Black, and Shuker (2020) showed that by ignoring cryptic mating failure (i.e., by assuming that all copulations involve sperm transfer), then paternity is strongly bimodal, with either high *P*_1_ or high *P*_2_. Taking cryptic mating failure into account, however, reveals the true picture of sperm competition and how paternity is assigned: a fair raffle with a key role played by copulation duration (see also Balfour, Armand, and Shuker 2024; Balfour, Corliss, and Shuker 2024; García‐González (2004) provides a thorough discussion of the role of mating failure—or ‘non‐sperm representation’—in the study of sperm competition).

Here we combine the study of sperm competition and mating failure, looking at the role of male and female size in determining both paternity and cryptic mating failure, with a particular focus on the role of copulation duration. Specifically, we asked how body size and cryptic male choice interact with sperm competition in this species (see also Balfour, Black, and Shuker 2020). We generated large and small size classes of males and females and allowed females to copulate with two different males on consecutive days, with all different size combinations of females and males represented. Females were the recessive pale colour morph, and we used both pale males and wild‐type males to assess paternity (Balfour et al. 2018). We hypothesised that body size influences how competitive a male is during sperm competition, but that female size should also influence sperm investment, thanks to cryptic male choice. Therefore, we predicted that when a female mated twice, male fertilisation success (measured as the proportion of offspring fathered by the second male to mate, or *P*_2_) would be affected by both the body size of the males involved and also by the body size of the female. Males should be willing to compete more and transfer more sperm to larger females via longer copulations, so the chance of mating failure should be less when females are larger. While the focus of this paper is post‐copulatory sexual selection, we also provide relevant data on pre‐copulatory sexual selection for completeness.

## 2 Methods

### 2.1 Study Organism and Husbandry

*Lygaeus simulans* is an aposematic seed bug found across Europe, alongside its sibling species *Lygaeus equestris* (Deckert 1985). Wild‐type *Lygaeus simulans* were collected in Tuscany, Italy, in 2008 and 2009. Bugs were maintained in continuous culture on an *ad libitum* diet of organic sunflower seeds, plus a water source, at 29°C on a 22:2 h light: dark cycle following standard protocols (e.g., see Balfour, Armand, and Shuker 2024 for further details). The pale colour morph of *Lygaeus simulans* began to appear in the population in 2012 and was subsequently isolated and a pale population was established using the same rearing techniques. The mutation segregates as a simple Mendelian locus, with the pale allele being recessive to the wild‐type allele (see Balfour et al. 2018 for further details of the pale morph).

### 2.2 Experimental Protocol

To obtain virgin females and males for the experiment, we moved late instar nymphs from the wild‐type or pale *L*. *simulans* populations to nymph boxes (20 × 10 × 8 cm plastic boxes) stocked with an *ad libitum* supply of sunflower seeds, a cotton‐bunged 25 mL tube of deionised water (changed weekly) and cotton wool for bugs to shelter in. We checked these boxes every 2–3 days for newly eclosed adults, which were then transferred to adult tubs (108 × 82 × 55 mm plastic tubs) using storksbill forceps, separated by sex and phenotype. This procedure is sufficient for males and females to remain virgin. These tubs were stocked with an ad libitum supply of sunflower seeds, a cotton‐bunged 7 mL tube of deionised water and a piece of cotton wool. No more than 10 individuals were housed in any given adult tub. All boxes and tubs were kept in the incubator in the same rearing conditions as the population boxes.

To generate the two female and male size classes for the experiment, we used the same measurement protocol as described in Balfour, Armand, and Shuker (2024) and Balfour, Corliss, and Shuker (2024). Briefly, virgin females and males were measured by gently placing them between two glass slides to hold the bug still. We then used a calibrated dissecting microscope fitted with an eyepiece micrometre and measured the body length from the end of the snout to the tip of the wings. Intermediate (or ‘medium’) sized bugs were sized ±half a standard deviation (SD) around the population mean (split by sex, as females are larger than males; females = 11.4–11.8 mm, males = 10.5–10.9 mm). Large males and females were therefore bugs larger than this medium class and small males and females were likewise smaller than these medium individuals. The means and SDs of males and females were taken from Balfour et al. (2018). (Note, medium‐sized females and males were then used in the experiment described in Balfour, Corliss, and Shuker 2024, and so were not discarded.) Individuals were measured 48–72 h prior to experimental trials and placed in same‐sex, same‐size category tubs with up to a maximum of 10 individuals in each tub, as described above.

The experimental trials ran across 2 days, with each focal pale female being paired first with a pale male on Day 1 for 6 h, and then a wild‐type male for 6 h on Day 2 (mate trials are described below). With our two female and male size classes, there were eight treatment combinations: LLL, LLS, LSL, LSS, SLL, SLS, SSL and SSS (with the first letter denoting the size of the female, the second letter denoting size of the Day 1 pale male and third letter the size of the Day 2 wild‐type male; L = large, S = small; note that for ease of exposition in the text below we talk about ‘large’ and ‘small’ individuals in the context of these experimental size classes). Bugs were paired up pseudo‐randomly with respect to body length (i.e., for treatment LLL, a large female was paired with a randomly selected large pale male on Day 1, and then with a randomly selected large wild‐type male on Day 2). Treatments were assigned based on the availability of different size categories of bugs (i.e., some weeks there were more females assigned to the treatment LSS than LLL due to limited availability of large males that week) so that over the course of the 10 weeks that the experiment was run, the sample sizes across treatments were as even as possible. The respective final sample sizes (i.e., for females which copulated on both days) were: *N* = 48, 68, 47, 57, 41, 91, 44 and 56. We always used pale males for Day 1, and wild‐type males for Day 2, as the experiment already contained eight treatment combinations and counter‐balancing at the level of male genotype would have doubled that, adding greater practical complexity to the experiment. However, previous data have shown that the effect of being the first or second male in similar sperm competition trials in terms of *P*_2_ outcome is independent of pale/wild‐type genotype (Balfour, Black, and Shuker 2020). However, we recognise that order effects are potentially confounded with male genotype to some extent, but such effects are not the focus of the current study.

The mate trials themselves were conducted as follows. On Day 1, we paired up virgin males and females pseudo‐randomly (for instance, for treatments LSS and LSL, on Day 1, we randomly selected a large female and paired it up with a randomly selected small male) in Petri dishes (55 mm diameter) and allowed them to interact and mate for up to 6 h. During the 6 h trial, we observed pairs by eye using scan sampling every 15 min and recorded whether they were engaged in copulation behaviour (yes/no) by seeing whether pairs were in the distinctive back‐to‐back copulatory position. Note, this positioning does not yet imply that male genitalia are fully deployed inside the female or that actual sperm transfer is taking place (see below). Scan sampling allowed us to observe multiple mate trials simultaneously. If pairs began copulatory behaviour but broke apart after two consecutive checks or less (i.e., < 30 min back‐to‐back) then they were left to re‐mate. This is because the shortest period of time required for successful insemination is 30 min (Gschwentner and Tadler 2000; see also Tadler, Nemeschkal, and Pass (1999) who showed in their study that the shortest copulation involving insemination lasted 71 min. Note that for all analyses throughout this paper, copulations < 30 min in length were not counted as a copulation, although we appreciate that important male–female interactions may still be occurring; our focus however is primarily post‐copulatory outcomes where sperm transfer at least has the potential to have occurred). If pairs broke apart after copulating for three consecutive checks or more (> 30 min) we then separated the pair, euthanising the male by placing it in the freezer at −18°C, and placed the female in an individual tub (108 × 82 × 55 mm plastic deli tub) with a cotton‐bunged water tube (7 mL) and 20–30 sunflower seeds. During the trials, as well as recording copulation duration, we also recorded the latency to mating (time to initiate copulation in the characteristic back‐to‐back position), again in terms of 15 min scans. At the end of the 6 h trial, all remaining pairs were separated, and males were euthanised while females were again placed in individual tubs. If pairs were still engaged in copulation, then they were separated by gently brushing the genitalia with a paintbrush. Focal females which did not engage in a successful copulation on Day 1 (back‐to‐back position for > 30 min) were discarded (euthanised at −18°C) as we were only interested in females which copulated successfully twice.

On Day 2, the same mating trial procedure was carried out, re‐using the focal females, pairing them again pseudo‐randomly with a male from the appropriate treatment. Again, only females which were observed in copula for three consecutive scans or more (> 30 min) were kept and returned to the incubator in their individual tubs while females which did not were euthanised.

During the Day 2 trials, each female's individual tub was checked for the presence of eggs (i.e., oviposition after Day 1). If any eggs were present, the female was given a fresh individual tub following the mating trial and the previous tub was frozen and discarded (*N* = 2). This meant that we only considered eggs and subsequent offspring that at least had the potential to have been fertilised by either the Day 1 or Day 2 male, depending on the particular insemination and sperm dynamics in any individual case.

We allowed females to lay eggs for a further 7 days and we then euthanised females at −18°C and checked tubs for the presence/absence of eggs, discarding any tubs without eggs. We then returned tubs to the incubator for a further week and then froze the tubs at −18°C for a minimum of 24 h before scoring tubs for the presence/absence of nymphs and counting any nymphs present according to colour morph. Since pale females were used, and since the pale colour morph is recessive while the wild‐type morph is dominant (Balfour et al. 2018), all pale nymphs were assumed to have been sired by the pale male which copulated on Day 1, and all wild‐type nymphs sired by the wild‐type male that copulated on Day 2. Previous data suggest that if the pale allele is still segregating in our wild‐type population stocks, then it is doing so at a very low frequency (Balfour et al. 2018), and so here all wild‐types are assumed to be homozygous at this locus. The *P*_2_ (second male paternity) was therefore calculated by dividing the number of wild‐type nymphs by the total number of nymphs present.

### 2.3 Statistical Analyses

We used one‐sample *z*‐tests to test whether *P*_2_ differed from equal paternity and whether the proportion of females that experienced *P*_2_ values of 0 or 1 differed from random. We used a Welch two‐sample *t*‐test to test whether copulation duration was longer on Day 2 than on Day 1, and a paired *t*‐test to test whether individual females copulated for the same duration on Days 1 and 2. We used Pearson's correlation to test whether there was a correlation between the copulation duration on Days 1 and 2 for individual females. To test whether mating failure depended on Day and/or male phenotype, we used a Chi‐squared test. Following previous analyses (Balfour, Black, and Shuker 2020), a Chi‐squared test was also used to explore whether mating failure was a female associated trait, i.e. did the observed number of double matings that resulted in 2 successes (nymphs sired from both males), 1 success (nymphs sired from one male), or 2 failures (no nymphs sired) differ from a random expectation.

We used quasibinomial generalised linear models (GLMs) to test the relationship between *P*_2_ and (i) copulation duration, (ii) the difference in copulation duration between Days 1 and 2, and (iii) female size class, (iv) male size class and (v) the interaction between these. We used binomial GLMs to test the relationship between female size, male size, and the interaction between these on (i) the likelihood of mating failure occurring and (ii) the likelihood of a pair engaging in copulation within the first 15 min of the trial. We also used a binomial GLM to test relationship between copulation duration and the likelihood that a male sired offspring. Finally, we used GLMs with a Gaussian distribution to test the relationship between female size class, male size class, and the interaction between these on (i) the copulation duration and (ii) the number of offspring sired. We also used binomial GLMs to explore (i) the relationship between female body size and the number of eggs laid and also (ii) the relationship between copulation duration and the number of nymphs sired. We used so‐called type II sums‐of‐squares throughout for the testing of terms in the models, with ‘*F*’ tests for the Gaussian and quasibinomial GLMs, and likelihood‐ratio (‘LR’) tests for the binomial GLMs, presented as *χ* ^2^ test statistics. As described in Balfour, Armand, and Shuker (2024) and Balfour, Corliss, and Shuker (2024), for analysis, pairs were only considered to have copulated if observed in copula for > 30 min (the minimum length of time for sperm transfer: Gschwentner and Tadler 2000).

## 3 Results

### 3.1 Pre‐copulatory Sexual Selection

**FIGURE 1:**
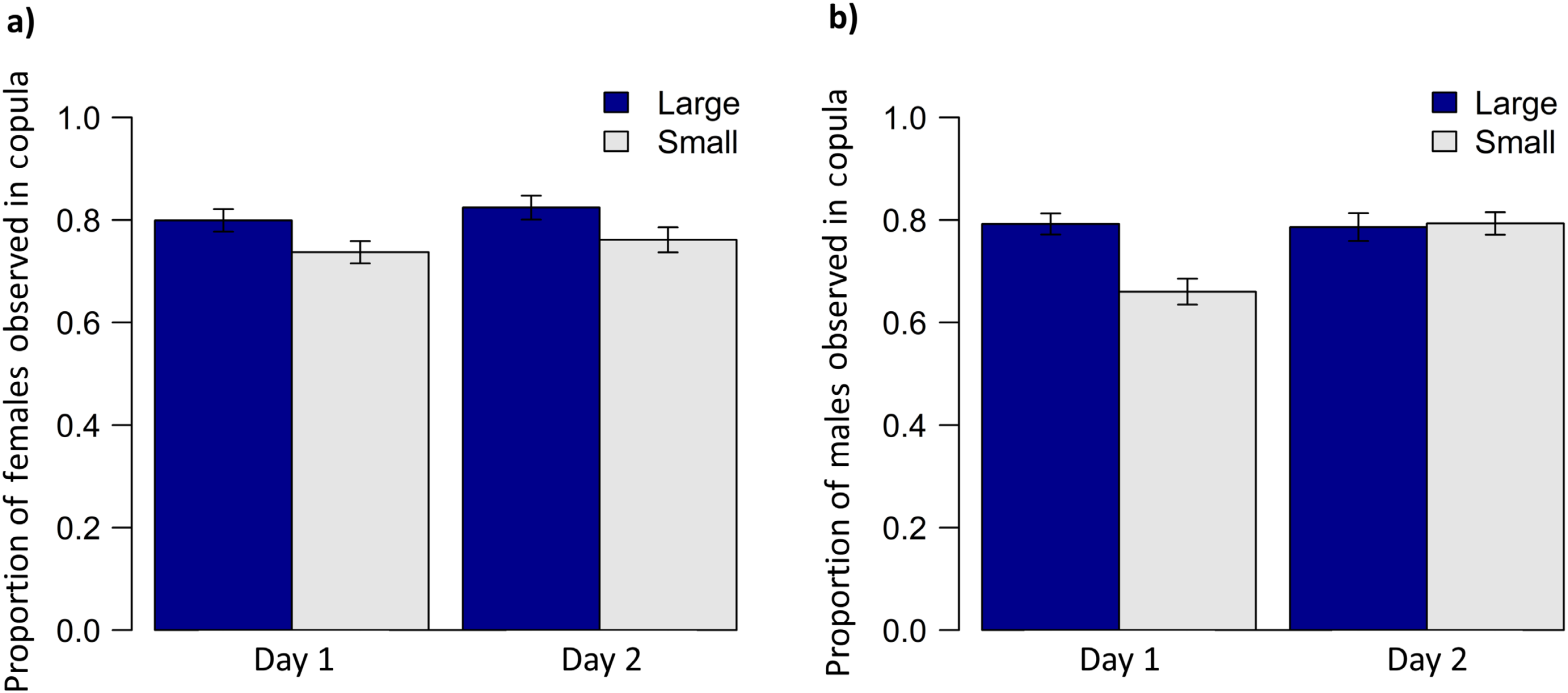
Pre‐copulatory sexual selection with respect to female and male size class. (a) The proportion of large (dark blue bars) and small (light grey bars) females that copulated on Day 1 (*N* = 334, 415) or Day 2 (*N* = 267, 305) and (b) the proportion of large (dark blue bars) and small (light grey bars) males that copulated on Day 1 (pale males, *N* = 395, 394) or Day 2 (wild‐type males, *N* = 229, 343; see main text for how size classes for males and females were generated). Error bars are standard errors.

In terms of pre‐copulatory sexual selection, 573 of the 749 females (76.5%) copulated for 30 min or more on Day 1. The females which copulated on Day 1 were then presented with a male on Day 2, of which 452 (78.9%) copulated for > 30 min. On Day 1, both large females and males were more likely to copulate (female size: $\chi_{1}^{2}$ = 4.5, *p* = 0.034; male size; $\chi_{1}^{2}$ = 3.98, *p* = 0.046; there was no interaction between male and female size: $\chi_{1}^{2}$ = 1.55, *p* = 0.213; Figure 1). On Day 2, which considers only females that had successfully copulated on Day 1, large females were only marginally and non‐significantly more likely to copulate ($\chi_{1}^{2}$ = 3.48, *p* = 0.062) and there was now no effect of male size ($\chi_{1}^{2}$ = 2.9, *p* = 0.234). However, there was a significant interaction, with pairings of large females and large males being more likely to copulate than any of the other three treatment combinations (87.2% vs. 79.5% or less: $\chi_{1}^{2}$ = 5.85, *p* = 0.016).

In terms of latency to mating, on Day 1, when considering all females that copulated on Day 1, and not just those that also copulated on Day 2, 53.8% of pairs copulated within the first 15 min, while on Day 2, 60.2% of pairs which copulated did so in the first 15 min. On Day 1, large females were far more likely to engage in copulation in the first 15 min than small females (61.4% vs. 47.1%: $\chi_{1}^{2}$ = 11.59, *p* = 0.0006), but there was no effect of male size ($\chi_{1}^{2}$ = 1.19, *p* = 0.276) nor any interaction ($\chi_{1}^{2}$ = 0.13, *p* = 0.715). For Day 2, all terms in the model were non‐significant (female size: $\chi_{1}^{2}$ = 0.30, *p* = 0.583; male size: $\chi_{1}^{2}$ = 1.82, *p* = 0.178; interaction: $\chi_{1}^{2}$ = 1.82, *p* = 0.177).

### 3.2 Post‐Copulatory Sexual Selection

In terms of post‐copulatory selection, we will first consider copulation duration. For the following analyses, only females that copulated on both Days 1 and 2 are considered. Overall, Day 2 pairs with non‐virgin females copulated for longer than the Day 1 pairs with virgin females (non‐virgin females = 244.4 ± 5.8 min; virgin females = 220.3 ± 5.4 min; Welch two‐sample *t*‐test: *t*_895.1_ = 3.04, *p* = 0.002). The same pattern is seen when comparing individual females on Days 1 and 2 (Paired *t*‐test: *t*_451_ = −3.40, *p* = 0.0007), and there was only a rather weak—if highly significant, thanks to the sample size—positive correlation between the copulation durations on each day for each female (Pearson's Correlation test: *r* = 0.2, *t*_450_ = 4.37, *p* < 0.0001).

Female size was strongly associated with copulation duration, with large females copulating for longer than small females on both Day 1 and Day 2 (Day 1: 245.7 ± 7.5 min vs. 196.2 ± 7.3 min; *F*_1,448_ = 22.4, *p* < 0.0001; Day 2: 263.6 ± 7.9 min vs. 226.2 ± 8.4 min; *F*_1,448_ = 10.06, *p* = 0.002). Male size did not influence copulation duration on either Day 1 or Day 2 though (Day 1: *F*_1,448_ = 0.11, *p* = 0.746; Day 2: *F*_1,448_ = 0.43, *p* = 0.514), and there were also no significant interactions between female and male size treatments on either day (Day 1: *F*_1,448_ = 0.38, *p* = 0.538; Day 2: *F*_1,448_ = 0.42, *p* = 0.515).

**FIGURE 2:**
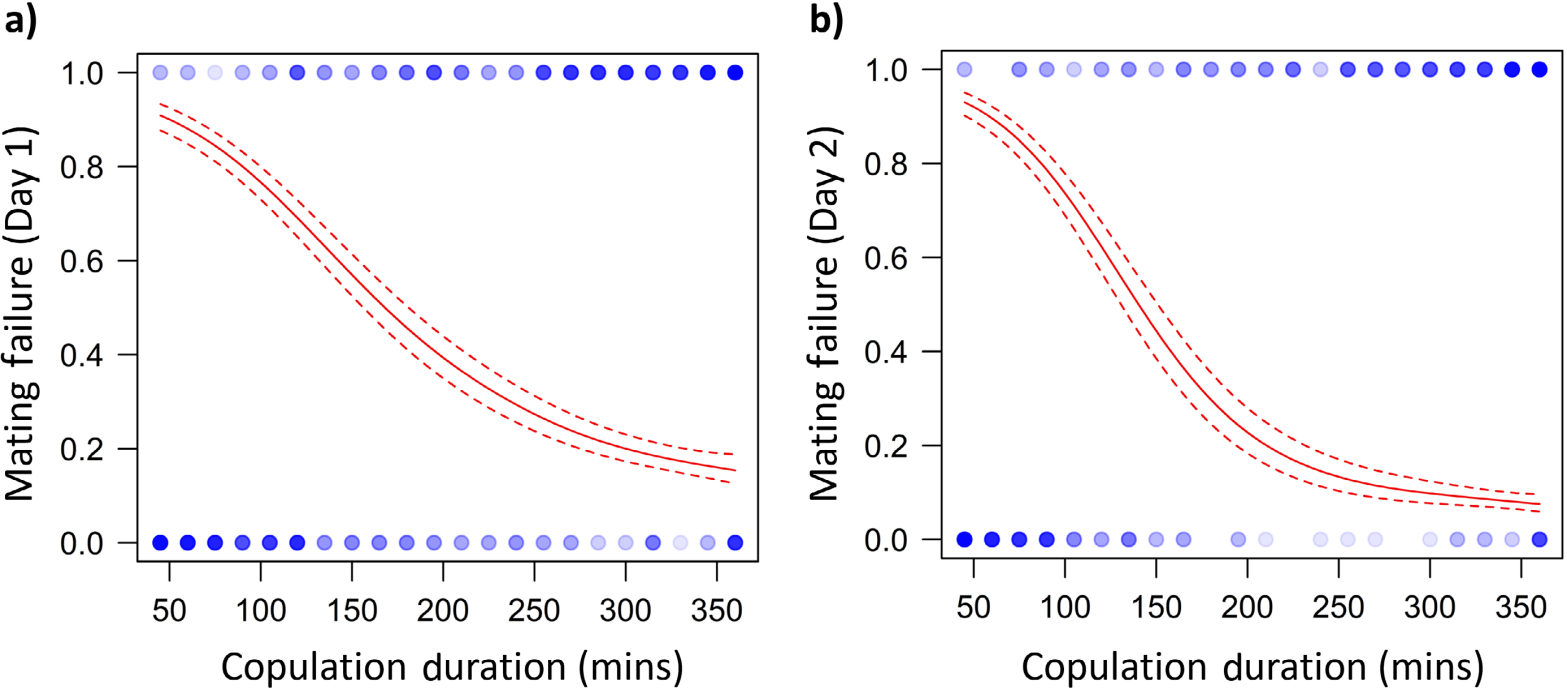
The relationship between copulation duration and mating failure (whether females had nymphs [0] or not [1]), for males which mated on (a) Day 1 and (b) Day 2, visualised as cubic splines (*N* = 452). Data are represented by circles, with the colour reflecting the number of individuals of the given size and mated state (darker = more replicates). Dashed lines indicate 1 standard error above and below the predicted line.

Copulation duration was strongly associated with (cryptic) mating failure. Copulation duration was positively associated with successful nymph production for both Day 1 and Day 2 copulations (scored as males successfully siring offspring: Day 1: $\chi_{1}^{2}$ = 158.1, *p* < 0.0001; Day 2: $\chi_{1}^{2}$ = 241.2, *p* < 0.0001; Figure 2).

Importantly, nearly all of the females that copulated across both days laid eggs (96%). However, large females were still more likely to lay eggs than small females (98.6% vs. 93.5%; $\chi_{1}^{2}$ = 8.41, *p* = 0.004), suggesting that either larger females were more developed in terms of their gravidity and willingness to lay eggs, or one of more of their mating partners contributed to a greater willingness to lay eggs (although we note the generally high level of oviposition of all the females). In total, 16.4% of these double‐mated females experienced complete mating failure and did not have any offspring at all (i.e., no eggs hatched to produce nymphs—we note here that both virgin and non‐virgin females can lay unfertilised eggs). When considering each mating event individually however (i.e., from the male perspective), the overall mating failure rate was 38.2%. In more detail, 43.6% of Day 1 pairings failed to result in any offspring sired by that male, whereas 32.7% of Day 2 pairings failed to result in offspring sired by the second male, a difference that was significant ($\chi_{1}^{2}$ = 10.8, *p* = 0.001).

Alongside copulation duration, female body size also had a major effect on the occurrence of mating failure. Large females were more likely to produce offspring from both Day 1 and Day 2 pairings than small females (Day 1: large females with offspring = 64.5%, small females = 48.7%; $\chi_{1}^{2}$ = 11.9, *p* = 0.0006; Day 2: large females with offspring = 73.2%, small females = 61.6%; $\chi_{1}^{2}$ = 7.06, *p* = 0.008). Mating failure was not associated with Day 1 or Day 2 male size treatment though (Day 1: $\chi_{1}^{2}$ = 1.27, *p* = 0.259; Day 2: $\chi_{1}^{2}$ = 0.363, *p* = 0.547). Moreover, there was no interaction between female size or male size on the chance of mating failure for either Day 1 or Day 2 copulations (Day 1: $\chi_{1}^{2}$ = 0.023, *p* = 0.880; Day 2: $\chi_{1}^{2}$ = 0.110, *p* = 0.740).

For those females which did not experience mating failure on Day 1, large females produced more nymphs than small females (39.4 ± 2.5 vs. 30.9 ± 2.2 nymphs; *F*_1,251_ = 6.13, *p* = 0.014). The effect of female size was only marginal on Day 2 (42.5 ± 2.1 vs. 36.9 ± 2.1 nymphs; *F*_1,251_ = 3.38, *p* = 0.067; note that females generally produced more nymphs on Day 2). Male size did not influence nymph production, either as main effects for Day 1 and Day 2 (Day 1: *F*_1,251_ = 0.124, *p* = 0.725; Day 2: *F*_1,251_ = 0.434, *p* = 0.510) nor via interactions with female size (Day 1: *F*_1,251_ = 0.640, *p* = 0.424; Day 2: *F*_1,251_ = 0.208, *p* = 0.649).

**FIGURE 3:**
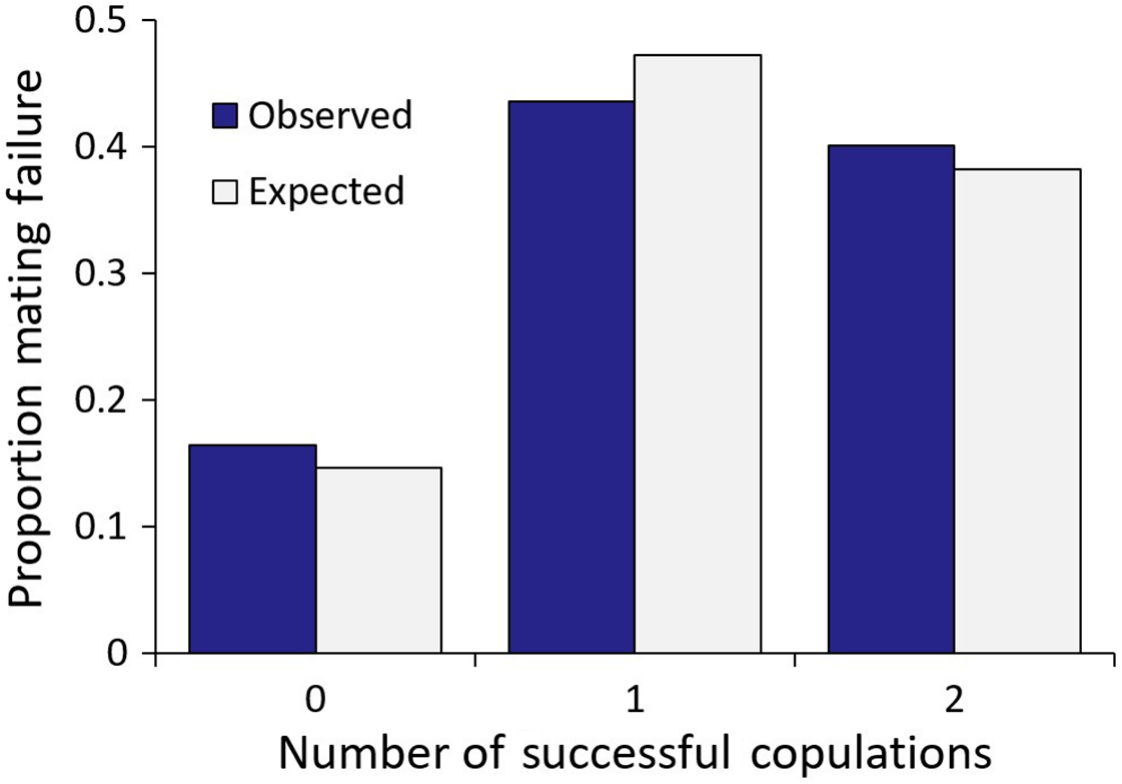
The distribution of observed (dark blue bars) and expected (light grey bars) successful copulations for females (*N* = 452). A copulation was deemed successful if nymphs of the same phenotype as the father were produced. The expected number of successful copulations was calculated from the overall proportion of mating failure of 0.382.

We also tested to see whether, *independently* of female size, some females were more or less likely to experience two failed matings (i.e., no nymphs produced), one successful mating (i.e., nymphs of one colour morph produced) or two successful matings (i.e., nymphs of both colour morphs produced), than expected by chance, calculated from the overall mating failure rate of 38.2%. We found that they did not, with the distribution of these possible outcomes not significantly different from random expectations ($\chi_{2}^{2}$ = 2.63, *p* = 0.269; Figure 3). Note, the results above show that mating failure is decidedly non‐random when female size is considered.

### 3.3 Patterns of Sperm Competition

**FIGURE 4:**
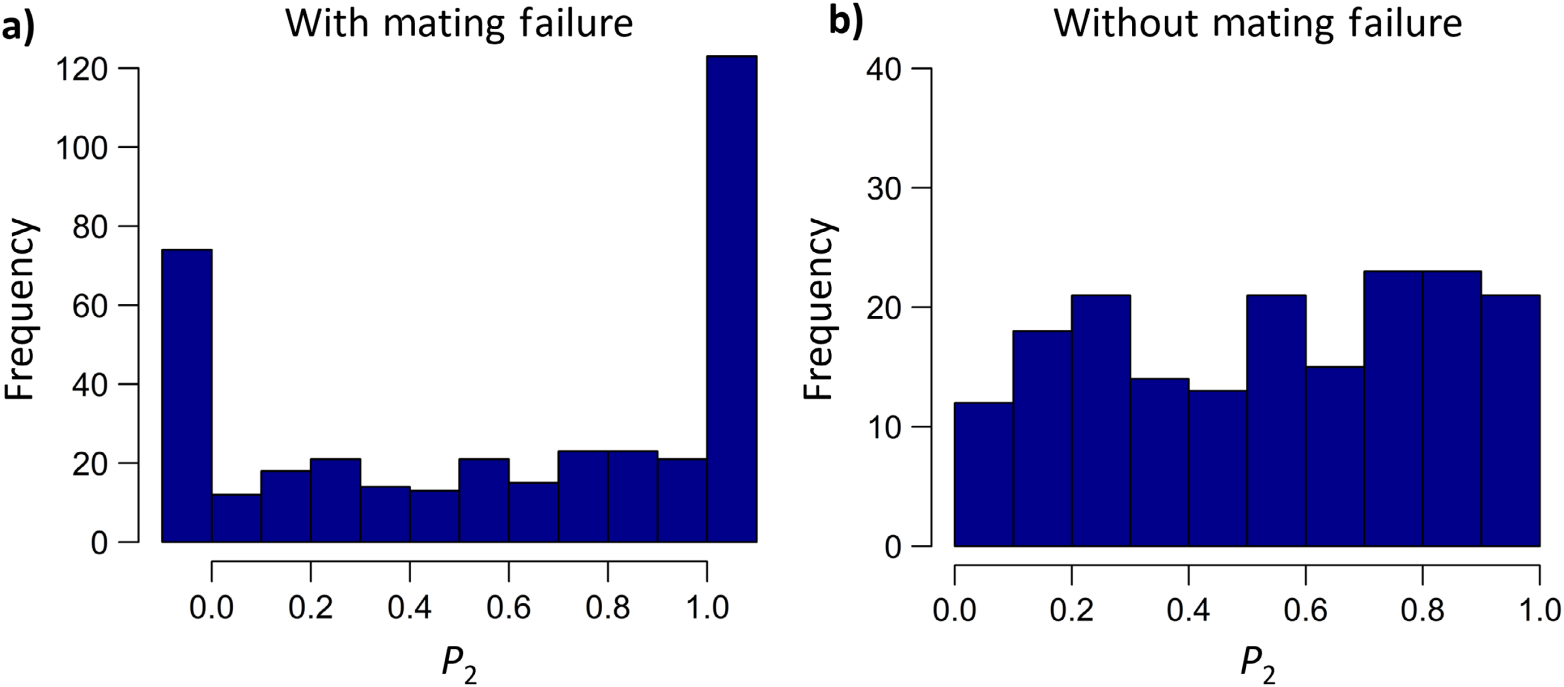
Distribution of *P*_2_ values across all treatments for females which (a) produced nymphs of at least one colour morph (*N* = 378), (b) produced nymphs of both colour morphs (i.e., without mating failure, *N* = 181). For (a) the first bin represents values of *P*_2_ = 0, i.e. these females produced nymphs only fathered by the Day 1 males. Conversely the final bin represents values of *P*_2_ = 1 where all offspring were fathered by the Day 2 male.

**FIGURE A1:**
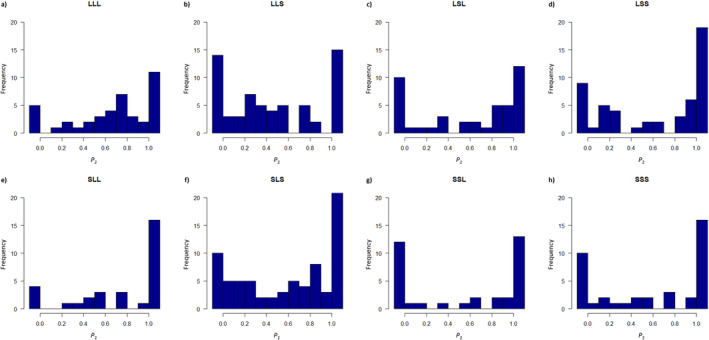
Distribution of *P*_2_ values across each treatment combination for females which produced nymphs of at least one colour morph. The first bin represents values of *P*_2_= 0, i.e., these females produced nymphs only fathered by the Day 1 males. Conversely the final bin represents values of *P*_2_ = 1 where all offspring were fathered by the Day 2 male. The treatment combinations are as follows (a) LLL, (b) LLS, (c) LSL, (d) LSS, (e) SLL, (f) SLS, (g) SSL and (h) SSS. The first letter denotes the size of the female, the second letter the size of the Day 1 male, the third letter the size of the Day 2 male, L = large, S = small (see main text for how size classes for males and females were generated). The respective sample sizes are; *N* = 41, 63, 43, 52, 31, 73, 35 and 40. For the mean estimates of *P*_2_ with and without mating failure, please refer back to Table 1 in the main text.

How do these patterns of mating outcomes shape patterns of sperm competition? Across all the females which produced nymphs, the overall *P*_2_ across treatments was 0.572 ± 0.003 (*N* = 378; Table 1; Figure 4, Figure A1). This was significantly different from an equal paternity of *P*_2_ = 0.5 (One‐proportion *z*‐test: $\chi_{1}^{2}$ = 112.5, *p* < 0.0001), but is not outside the predicted range of *P*_2_ = 0.4–0.6 for a random sperm mixing mechanism (García‐González 2004; Balfour, Black, and Shuker 2020; see Section 4). In general, the *P*_2_ was very bimodal, with the majority of values falling into the category of either *P*_2_ = 0 or *P*_2_ = 1 (Figure 4a). For these double‐mated females that produced only one nymph morph (*N* = 197), the proportion of females that experienced *P*_2_ values of 0 or 1 differed from random, with females more likely to experience a value of *P*_2_ = 1 rather than a value of *P*_2_ = 0 (62.4% vs. 37.6%; $\chi_{1}^{2}$ = 5.08, *p* = 0.024). When removing the effects of mating failure by only including females which produced nymphs of both colour morphs (*N* = 181), the *P*_2_ across all treatments was 0.550 ± 0.005, which again differed significantly from equal paternity, but was also no longer bimodal ($\chi_{1}^{2}$ = 112.5, *p* < 0.0001; Table 1; Figure 4b).

**TABLE 1:** Paternity estimates (as the proportion of nymphs sired by the second male to mate, or *P*_2_ ± standard error: SE) for each treatment and all treatments combined for (i) all females that had nymphs of one or more colour morphs (with mating failure) and (ii) only females that had nymphs of both colour morphs (without mating failure).

Treatment	With mating failure			Without mating failure		
	*N*	*P*_2_	SE	*N*	*P*_2_	SE
LLL	41	0.577	0.010	25	0.611	0.012
LLS	63	0.459	0.008	34	0.435	0.011
LSL	43	0.574	0.010	21	0.621	0.012
LSS	52	0.622	0.008	24	0.554	0.012
SLL	31	0.743	0.011	11	0.636	0.018
SLS	73	0.578	0.008	42	0.492	0.010
SSL	35	0.518	0.013	10	0.686	0.021
SSS	40	0.595	0.011	14	0.617	0.018
All treatments	378	0.572	0.003	181	0.550	0.005

Patterns of paternity were largely independent of female and male body size treatment combinations (female size: *F*_1,370_ = 2.13, *p* = 0.145; Day 1 male size: *F*_1,370_ = 0.41, *p* = 0.522; Day 2 male size: *F*_1,370_ = 1.22, *p* = 0.270). There was however a slight interaction between female size and Day 1 male size (*F*_1,370_ = 4.38, *p* = 0.037) with *P*_2_ being higher if the male and female were of a different size. There was also an interaction between Day 1 male size and Day 2 male size (interaction: *F*_1,370_ = 5.41, *p* = 0.021), with *P*_2_ being higher when both males were in the same size category. There was no interaction between female size and Day 2 male size (*F*_1,370_ = 0.06, *p* = 0.810), nor a three‐way interaction between female and male body size treatments (*F*_1,370_ = 0.29, *p* = 0.591).

When we exclude replicates with mating failure, and so look at replicates where both males contributed to the offspring, then there was again no effect of female size (*F*_1,373_ = 1.37, *p* = 0.242) and this time all interaction terms were non‐significant (*p* > 0.3). However, there was a marginally non‐significant effect of Day 1 male size on *P*_2_ (*F*_1,373_ = 3.17, *p* = 0.077), whereby there was a tendency for large pale males to gain a greater share of the paternity than small pale males (*P*_1_ = 0.48 vs. *P*_1_ = 0.40). There was also a significant effect of Day 2 male size on *P*_2_ (*F*_1,373_ = 7.57, *p* = 0.007), with large wild‐type males on the second day being more likely to gain a greater share of the paternity than small wild‐type males (*P*_2_ = 0.63 vs. *P*_2_ = 0.50).

Perhaps most crucially, for all double‐mated females that produced nymphs, there were clear relationships between *P*_2_ and (i) Day 1 copulation duration (*β* = −0.011 ± 0.003, *F*_1,374_ = 123.86, *p* < 0.0001) and (ii) Day 2 copulation duration (*β* = 0.009 ± 0.003, *F*_1,374_ = 165.33, *p* < 0.0001), but no interaction (*F*_1,374_ = 0.18, *p* = 0.674). In other words, the longer the copulation duration on Day 1, the lower the *P*_2_ (i.e., the male on Day 1 gets more paternity, so *P*_1_ is high, *P*_2_ is low), and the longer the copulation duration on Day 2, the higher the *P*_2_ (the male on Day 2 gets more paternity). The same pattern held true when excluding mating failure and considering females which produced offspring of both nymph morphs, hence values of *P*_2_ = 0 and *P*_2_ = 1 were excluded (Day 1 copulation duration: *β* = −0.008 ± 0.006, *F*_1,177_ = 10.63, *p* = 0.001; Day 2 copulation duration: *β* = 0.00018 ± 0.005, *F*_1,177_ = 15.16, *p* = 0.0001; interaction: *F*_1,177_ = 0.747, *p* = 0.389).

**FIGURE 5:**
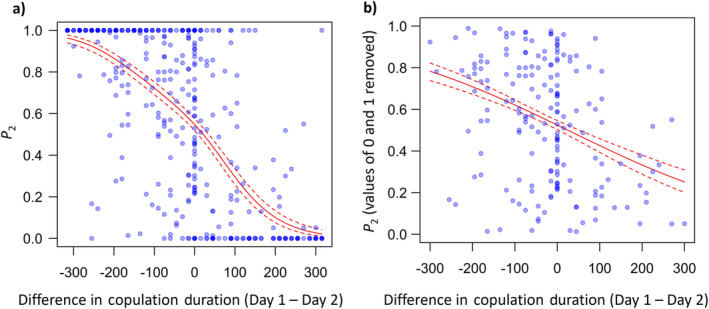
Paternity (*P*_2_) is influenced by the difference in copulation duration between Day 1 and 2 (all treatments combined). A negative value for difference in copulation duration indicates that the second male copulated for longer than the first male, while for positive values the opposite is true. (a) All females which produced nymphs of at least one colour morph (*N* = 378) and (b) females which only produced nymphs of both colour morphs and hence did not experience any mating failure (*N* = 181). Data are represented by circles, with the colour reflecting the number of individuals with the given copulation duration and *P*_2_ (darker = more replicates). Dashed lines indicate 1 standard error above and below the predicted line (solid line).

Finally, we considered the difference in copulation duration between Days 1 and 2 and the outcome on paternity. There was a significant effect of the difference in copulation duration on the *P*_2_ outcome for all double‐mated females that had nymphs, with whichever male mated for longer gaining a higher share of the paternity (*β* = −0.009 ± 0.0008, *F*_1,376_ = 269.45, *p* < 0.0001; Figure 5a). The same relationship was true when females that experienced mating failure with one male were excluded (*β* = −0.004 ± 0.0008, *F*_1,179_ = 24.01, *p* < 0.0001; Figure 5b).

## 4 Discussion

Understanding how different processes of sexual selection interact is crucial for understanding the role of sexual selection in phenotypic evolution (Hunt et al. 2009). Here we have explored how different processes of sexual selection play out in the seed bug *Lygaeus simulans*. In terms of pre‐copulatory sexual selection, large males and females were more likely to mate on Day 1 (less so on Day 2; Figure 1), suggesting some degree of mutual mate choice. These patterns have been shown before in *L*. *simulans* but are not consistent across studies (see Section 1), as indeed they were not consistent across Days 1 and 2. As we have manipulated the size ranges of males and females, perhaps we have exaggerated the opportunity for mate choice to occur. However, given the sample sizes, the effect sizes are still clearly small, and so it looks as though pre‐copulatory sexual selection for male and female size in this species is at best a rather weak form of selection.

In terms of post‐copulatory sexual selection, we shall first consider sperm competition. For double‐mated females, the patterns of paternity fit with those expected under random sperm mixing (Parker, Simmons, and Kirk 1990; Simmons 2001; García‐González 2004). In addition, whether or not we take mating failure into account clearly influences the pattern of paternity (García‐González 2004; Balfour, Black, and Shuker 2020; Figure 4), but what emerges is that copulation duration is a strong determinant of male paternity. This is true for males in either the ‘defensive’ role in sperm competition (i.e., the first male), or in the ‘offensive’ role (i.e., the second male). The role of copulation duration is most clearly shown in terms of the effect of the difference in copulation durations between first and second males and the resulting level of *P*_2_. Relatively longer copulations lead to higher paternity for both first and second males (Figure 5).

In *L. simulans* there is an initial period of copulation where there is no sperm transfer (around 30–40 min) during which time the male navigates the female reproductive tract with his processus gonopore (Gschwentner and Tadler 2000; Dougherty et al. 2015). Following this, there is a period of sperm transfer. Extremely long copulations seen in the sister species *Lygaeus equestris* of up to 24 h are expected to be a form of mate guarding (Sillén‐Tullberg 1981) and there is evidence that sperm transfer does not continue for the whole duration of a copulation. However, during the early stages of copulation, it is at least plausible that copulation duration is positively correlated with the number of sperm transferred. The distribution of *P*_2_ values was very bimodal, with many values of *P*_2_ equalling 0 or 1, which we assume to be caused by cryptic mating failure, and hence insemination failure (Greenway, Balfour, and Shuker 2017; Balfour, Black, and Shuker 2020). It is possible that in some of these cases, sperm was transferred by both males and only the sperm of one male was utilised. However, we expect there to only be a very small number of such cases as the mating failure rate for individual males (38.2%) falls in line with what we have found previously in this species for single matings (see above).

Interestingly, we found that values of *P*_2_ = 1 were more common than values of *P*_2_ = 0. Previously we have found that males are more likely to copulate with once‐mated females compared to virgins (Balfour, Black, and Shuker 2020). Perhaps it is easier to navigate the reproductive tract of already mated females, and so maybe it is easier for males to get their processus gonopore to the correct location in the female reproductive tract to transfer sperm (see Gschwentner and Tadler 2000 and Dougherty et al. 2015 for more details on the process of copulation in this species). Given that males are more likely to copulate with once‐mated than virgin females (Balfour, Black, and Shuker 2020) it does seem that males are able to tell the mating status of a female—though note this does not rule out the possibility that once‐mated females are just a more willing to copulate subset of females, compared to the whole virgin cohort, and so are more likely to mate twice. When we excluded cases of likely cryptic mating failure, and therefore cases when *P*_2_ = 0 or 1, there were no clear peaks in the data and *P*_2_ values tended to be uniformly distributed (as seen in Balfour, Black, and Shuker 2020). Again, this very much suggests that random sperm mixing is occurring.

The effects of male and female size on paternity were slightly more complicated, although less so when excluding females who had experienced mating failure in one of their two episodes of copulatory behaviour. The general pattern is that larger males gained more paternity, especially when copulating on Day 2 (Table 1, Figure A1). Therefore, the outcome of sperm competition seemed to favour both males which copulated for the greatest duration, and larger males. Given random sperm mixing, this could be because larger males are able to transfer more sperm per unit time compared to smaller males, plus longer copulations in and of themselves may result in a greater number of sperm being transferred. Similar patterns have been found in a range of other species, including the green‐veined white butterfly *Pieris napi* (Bissoondath and Wiklund 1997), and this has been suggested to be because body mass in this species correlates with the amount of ejaculate transferred (Wiklund and Kaitala 1995). Moreover, in the stone crab *Hapalogaster dentana*, although small and large males transferred the same amount of sperm to small females, large males increased the number of sperm transferred to large females whereas small males did not (Sato and Goshima 2007). Small males may be limited in how much sperm they can produce relative to larger males, resulting in only larger males being able to increase their ejaculate size when copulating with larger females which are more fecund (Sato and Goshima 2007). Although *L. simulans* female size did not seem to affect paternity outcome in our experiment, it does seem plausible that larger males would be able to transfer more sperm than smaller males. We have also recently shown that large males sire a greater number of nymphs than small males when they copulate with large females, again suggesting that large males can transfer a greater number of sperm and that there is cryptic male choice occurring via copulation duration and ejaculate investment (Balfour, Armand, and Shuker 2024). However, we will return to male ejaculation strategies shortly, after considering mating failure.

As outlined in the Section 1, cryptic mating failure is common in this species, with many apparent copulations failing to involve successful sperm transfer and offspring production (Greenway, Balfour, and Shuker 2017). Cryptic mating failure in *L*. *simulans* is most commonly negatively associated with female body size, with larger females being more likely to receive and/or accept sperm from a male. We have recently argued that this pattern is best explained by cryptic male choice for larger females (Balfour, Armand, and Shuker 2024; see also Arnqvist 2014; Aumont and Shuker 2018). Here we see the same pattern. Large females were more likely to avoid mating failure and receive sperm from each male they copulated with compared to small females. There was no effect of male size on mating failure. Moreover, large females were more likely to lay eggs, produced more nymphs, and also copulated for longer. Again, there were no effects of male size. Taken together, these data support the hypothesis that males prefer to inseminate larger females, and so copulate for longer and gain fecundity benefits from doing so.

The data also fit with copulation duration being the key mechanism by which cryptic male mate choice occurs in this species, with males firstly gaining whatever information criteria they are using for making their ejaculation decision (information we are still unclear about, apart from its presumed relationship to female body size), and then inseminating or not, with these processes together leading to longer copulations with preferred females. This fits with the literature more generally, with a meta‐analysis by Kelly and Jennions (2011) demonstrating that across different taxa, males transferred larger ejaculates and copulated for longer with higher‐quality females. It is also worth noting that we may well be underestimating the extent of cryptic male choice, as we excluded pairs in the back‐to‐position for less than 30 min in our definition of copulation.

Following encouragement from one of our reviewers, here we engage in some brief speculation about why males may engage in the kind of cryptic male choice we are arguing for. In particular, what seems unusual from how we normally think about mating systems is a male managing to engage genitalia with a female and then failing to proceed to pass sperm. As just mentioned, the data from this experiment, and others cited above, suggest that larger females are more likely to receive sperm. Previously, work on post‐copulatory sexual selection in this species, and its closely related sister species *L*. *equestris*, had focused more on cryptic female choice (following the influential synthesis by Eberhard 1996). For instance, there is evidence across different studies for stabilising selection on male genitalia length in *L*. *simulans* (Tadler, Nemeschkal, and Pass 1999; Dougherty et al. 2015), presumably due to the interactions between male genitalia and the female reproductive tract, perhaps because of the latter's length and/or structure.

For cryptic female choice to explain the association with female size and insemination success, we still a need a male phenotype that is being preferred by females (i.e., the target of choice) that is also correlated with female size. Larger females may be more willing to mate than smaller females or require more sperm than smaller females, but for this to be associated with cryptic female choice, we still need a male phenotype as a non‐random intermediary, which to date has not been identified, except in terms of the stabilising selection on male genitalia. On the other hand, female–female competition for access to male gametes may be such that larger females are more willing to mate and obtain sperm than smaller females, but in doing so do not exert any selection on males (i.e., male insemination success is effectively random with respect to male phenotype). There has been a welcome increase in the recognition of female–female competition for gametes, and hence sexual selection on females (Shuker and Kvarnemo 2021 and references therein). However, while female–female competition for gametes is perhaps plausible, for a species as polyandrous as *L*. *simulans*, where both males and females re‐mate frequently, it is not clear that sperm are limiting enough for females to come into competition for them or why larger females respond more than smaller ones to this competition.

Instead, it is possible that—as alluded to above—males can gain some information about females once mating has commenced that they are less able to gain prior to pairing up, for instance about female size and/or potential fecundity. As yet, we do not know what that information is—except that it should be strongly correlated with female size—nor do we know how they are obtaining it, although we do know that it appears to influence copulation duration most strongly. Clearly, a mix of behavioural and physiological work will be needed to further unpick both male and female sources of variation in post‐copulatory outcomes in this species.

Some further circumstantial evidence supportive of male choice comes from the distribution of mating failure across females *independently* of female size. The distribution of mating failure was not significantly different from a random expectation, as the proportion of females that had offspring from one, both, or neither of the males they copulated with did not differ from the expected proportions if mating failure was random (this is in contrast to previous findings where a weak association was found: Balfour, Black, and Shuker 2020). Therefore, this suggests that the pattern of small females being more likely to fail to produce offspring is due to male choosiness and not just due to some individual females being more susceptible to mating failure. Note that the results presented earlier clearly show that female mating failure is very much *non‐random* when we take female size class into account, however.

How do sperm competition and cryptic male choice interact? Generally, female size does not seem to have much impact on patterns of male paternity, in terms of sperm competition outcomes when males do inseminate females. However, female size does have a strong effect on whether insemination takes place in the first place. This suggests that different components of post‐copulatory sexual selection—sperm competition on males and cryptic male choice on females—do not interact with each other to a great extent. There was a very slight interaction between female size and Day 1 male size in terms of paternity, whereby the *P*_2_ was higher if the male and female were of a different size. However, this was only true when females which experienced *P*_2_ values of 0 and 1 were included (i.e., mating failure was included) in the data set.

However, a further possible interaction between sperm competition and cryptic male choice comes from the fact that males might expect larger females to represent arenas of greater sperm competition risk, given that they are the preferred mates of males generally. Therefore, they could choose to invest more in these females in order to try and outcompete rival males. In doing so, they may be more likely to pass sperm, or pass more sperm to these females, and so the overall mating failure rate in large females may be lower. Our data fit this scenario as well. Male strategic sperm allocation is now a very well‐developed field (Simmons 2001; Wedell, Gage, and Parker 2002; Kelly and Jennions 2011; Turnell, Shaw, and Reeve 2018; Bretman et al. 2023), although its links with cryptic male choice perhaps remain less explored (or maybe it would be better to say that strategic sperm allocation is not always appreciated as a potential mechanism of cryptic male choice; see Ramm and Stockley 2014). Strategic sperm allocation moves from being merely an adaptive response, to male–male sperm competition, to a mechanism of male mate choice, when the female phenotype influences male insemination decisions non‐randomly and some females are more likely to get access to preferred male gametes than others (Aumont and Shuker 2018; Shuker and Kvarnemo 2021). It would therefore be interesting to test whether males respond differently to females of different sizes, in terms of copulation duration, and mating failure outcome, when in the presence or absence of rival males. This could again further our understanding of the context‐dependent nature of sperm competition and cryptic male choice, in *L*. *simulans* and beyond. Finally, as noted by one of our reviewers, our study made use of pale and wild‐type genotypes to study post‐copulatory sexual selection in this species. A further task will be to develop alternative molecular genetic techniques to ensure the results presented here and elsewhere on *L*. *simulans* sperm competition generalise across genetic backgrounds.

In conclusion, our results show that sperm competition outcomes in *L. simulans* are primarily driven by copulation duration, with longer copulations leading to greater paternity share for a male. In line with previous findings, we again confirm that larger females are more likely to produce offspring than smaller females, as predicted by cryptic male choice for large females. This effect was again linked to copulation duration, with longer copulations less likely to lead to cryptic mating failure. While larger males tended to be more successful in sperm competition, especially if copulating second, female size had little effect on paternity, suggesting that cryptic male choice and sperm competition are acting relatively independently in this species.

## Author Contributions

**Vicki L. Balfour:** conceptualization (equal), data curation (lead), formal analysis (lead), investigation (equal), methodology (equal), project administration (equal), supervision (supporting), writing – original draft (lead), writing – review and editing (equal). **Mélissa Armand:** data curation (equal). **David M. Shuker:** conceptualization (equal), funding acquisition (lead), methodology (equal), resources (lead), supervision (lead), writing – original draft (equal), writing – review and editing (equal).

## Conflicts of Interest

The authors declare no conflicts of interest.

## Data Availability

The research data supporting this publication can be accessed at https://doi.org/10.17630/00bf7356‐f57d‐4353‐96bd‐22854fea1d65.
